# Simulating virtual images in optical trap displays

**DOI:** 10.1038/s41598-021-86495-6

**Published:** 2021-04-06

**Authors:** Wesley Rogers, Daniel Smalley

**Affiliations:** Department of Electrical and Computer Engineering, Brigham Young University, Provo, UT 84602 USA

**Keywords:** Displays, Engineering, Optics and photonics, Physics

## Abstract

Optical trap displays (OTD) are an emerging display technology with the ability to create full-color images in air. Like all volumetric displays, OTDs lack the ability to show virtual images. However, in this paper we show that it is possible to instead *simulate* virtual images by employing a time-varying perspective projection backdrop.

## Introduction

In 2018, the authors presented a new platform for volumetric display using photophoretic trapping^1^. This display operates by first confining a particle in a photophoretic trap which then drags the particle through every active image point in a freespace volume. In the current prototype, this volume is typically 1 cm^3^. As the particle is dragged through space, it is illuminated by visible lasers to form an image by persistence of vision. The volume must be redrawn more than ten times a second to be above the flicker rate of the eye. The most immediate current challenges to bring the display into the realm of practicality are (1) to scale the display volume from 1 cm^3^ to greater than 100 cm^3^ and (2) to employ parallel traps^2^ to address the fundamental incapacity of freespace volumetric displays to create virtual images. Our efforts to address the former challenge will be reported in another publication. Instead, this work focuses on the latter challenge, the challenge of simulating virtual images with freespace volumetric hardware.

Also, photophoretic optical trap displays have a fluid drawing volume, which means that it is theoretically possible to create images that are much larger than the display itself. However, there are practical limits to how large a volumetric image may become. Volumetric images are defined as having image points co-located with physical point scatterers^3^. The physicality of these volumetric points gives them perfect accommodative cues (because the viewer is focusing on a physical object). This definition requires that volumetric images be composed of real image points that must exist only in a finite drawing volume. So, to display an optically correct volumetric image of the moon seen through a window would require the OTD display to be scaled to *astronomical* proportions. This situation is not unlike that of a movie set or theatrical stage, where props and players must occupy a fixed space even when trying to capture a scene meant to occur outdoors or in outer space. In the theater, this limitation is mitigated by including a flat backdrop that contains pictorial 3D cues such as a road winding to a point (perspective cues) or mountains eclipsing one another (occlusion cues) as they fade (atmospheric cues) into the distance in order to create the sense of enlarged space. In a modern theater production, using projections for backdrops, motion can also be used to simulate parallax. This is effective because the background depicts sites at distances where the focus cues like accommodation and vergence would not be dominant. This approach could also be used to simulate virtual images. Volumetric images share these challenges and could share these solutions. With purely real volumetric image points, freespace volumetric displays will be forever confined to the drawing volume. What is needed is a ‘backdrop’ for volumetric displays.

**Figure 1:**
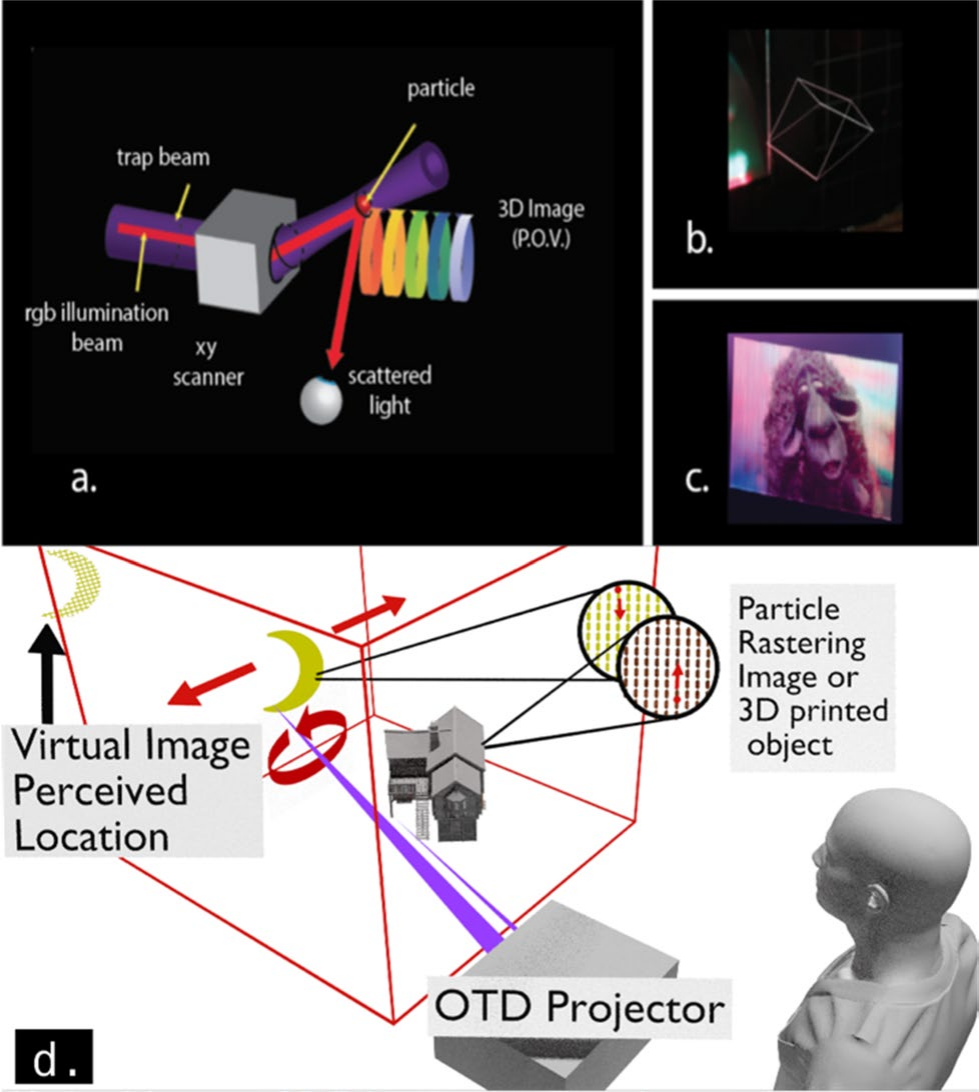
OTD display and simulated virtual images concept. (**a**) Optical trap display (OTD). (**b**) 3D vector, long exposure, image drawn by OTD. (**c**) Flat, rastered, long-exposure image drawn by OTD; content from *Cosmos Laundromat,* (CC) Blender Foundation|gooseberry.blender.org. (**d**) Simulated virtual image concept with flat moving/rotating plane at the back of a draw volume filled with real images/objects such as 3D OTD images or 3D printed objects.

In this work, we apply and extend the backdrop analogy to simulate virtual images in a photophoretic optical trap display (OTD). OTDs can draw flat and 3D structures in air (Fig. 1b,c). It is possible to draw an image at the edge of the drawing volume and modify its apparent parallax while tracking the viewer to create an image that behaves optically as if it is located behind the display volume (Fig. 1d). This technique is referred to in the field of computer graphics as ‘perspective projection’ and it is achieved in OTDs by modifying the scale, shape, and parallax of the content on a background image plane as the viewer moves. The plane may also rotate to face the viewer in situations where the plane is finite (not spherical to encompass the viewer), see Visualization 3. Cossairt^2^ points out the limitation that all image points must lie along a line extending from the observer through the display volume**.** The points that the user perceives in the back plane are no longer volumetric because they no longer coincide with physical scatters, so they lose the attribute of perfect accommodation^2,4–6^, but they gain the ability to dramatically increase the perceived size of the image volume. Using a perspective projection, an OTD can simultaneously generate both real volumetric image points for the foreground and simulated, non-volumetric image points for the background, greatly expanding the usefulness of the OTD platform.

The small display volume makes the current prototype of limited utility, and we will have to wait for larger parallel prototypes to test many display-level characteristics^7^. However, even in this small volume, it is possible to create a simple proof-of-concept experiment for simulating virtual images. We also feel compelled to do so as quickly as possible because the inability to create virtual images is so fundamentally limiting to the entire freespace family. Therefore, the result of even this small-scale experiment literally and dramatically *expands* the application space for all freespace volumetric displays capable of angular scatter control.

## Theory

### Optical trap displays

Optical trap displays operate by confining one or more particles in a photophoretic trap. Particles of many different materials, sizes, and geometries have been demonstrated in optical traps^8,9^. This paper uses cellulose particles estimated at 10 μm^1^. When the trap is moved, the particle is dragged along with it. The particle is moved through all of the image points in succession. When the particle reaches an image point, it is illuminated with a combination of red, green, and blue light. The particle moves through every point in the image several times a second creating an image by persistence of vision (see Fig. 1a). The persistence of vision refresh rate (> 10 frames/s) could be considered a lower bound for creating a convincing ‘backdrop’. The higher the resolution and the refresh rate of the system, the more convincing this effect can be made as the user will not be able to perceive updates to the imagery displayed to them and at sufficient resolution will have difficulty distinguishing display image points from real world image points. We should note that drawing a frame, in the context of this paper, refers to the trap’s traversing of a vector path through a collection of image points. In this ‘vector mode,’ the number of points drawn and resulting update rate is image-dependent.

### Perspective projection

One of the most general forms of perspective is ray tracing where the observer or camera is considered as a single point E = (x_0_, y_0_, z_0_), the image point to be displayed X = (x, y, z), and the plane on which to display P. Finding the intersection of the line EX with the plane P gives the pixel coordinate of the point X. The perspective projection can be defined by the following matrix relationship for a plane P perpendicular to the line EO where O is the origin:

1 $${\text{S}} = \left[ {\begin{array}{*{20}l} { - {\text{r}}^{2} } \hfill & {{\text{r}}^{2} {\text{x}}_{0} } \hfill & 0 \hfill & 0 \hfill \\ { - {\text{rx}}_{0} {\text{z}}_{0} } \hfill & { - {\text{ry}}_{0} {\text{z}}_{0} } \hfill & {{\text{r}}{\uprho}^{2} } \hfill & 0 \hfill \\ 0 \hfill & 0 \hfill & 0 \hfill & {{\text{r}}{\uprho}} \hfill \\ { - {\uprho} {\text{x}}_{0} } \hfill & { - {\uprho} {\text{y}}_{0} } \hfill & { - \rho {\text{z}}_{0} } \hfill & {{\text{r}}^{2} {\uprho}} \hfill \\ \end{array} } \right]$$

2 $$r = \sqrt{\left({x}_{0}^{2}+{y}_{0}^{2}+{z}_{0}^{2}\right)}$$

3 $$\rho = \sqrt{{x}_{0}^{2}+{y}_{0}^{2}}$$

The perspective projection matrix is designed to project a scene from space to the plane^10,11^. This allows for the representation of 3D points using a 2D surface which preserves all pictorial cues for a specific 3D observation point. For a video example at several depth planes, see “Supplemental Document” and Visualization 1. By using a dynamic observation point, co-located with a real observer or a simulated observer, such as a camera, and an updating image plane, the visual cues of 3D image points can be achieved for the pictorial cues and motion parallax cues.

## Experiment

**Figure 2:**
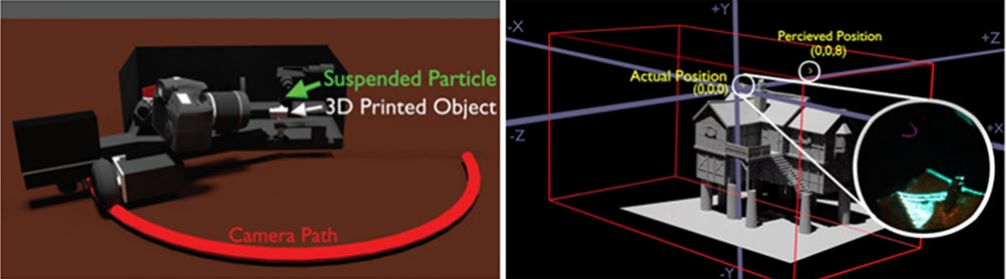
Experiment setup. (**a**) An OTD display projects a flat moon image at the back of a draw volume that contains a 3D printed house. The image is updated at persistence of vision frame rates (12 frames/s) using the perspective projection based on expected camera location. (**b**) A close-up of the house position, moon position, and perceived moon position in 3D space.

To demonstrate simulated virtual images using modified parallax (perspective projection), we drew a flat (2D) OTD image of the moon at the back face of our drawing volume. This plane, in turn, sat at the front face of a 3D printed miniature of a house (See Fig. 2b). A camera was placed on a rotating arm (See Fig. 2a). The OTD image of the moon was drawn and redrawn at persistence of vision rates (12 frames/s). In previous studies^8^, the OTD has been shown to have a maximum demonstrated velocity of 1.8 m/s. The number of voxels per second drawn for the experiments in this work is approximately 10 k voxels/s. Millimeter-scale vector images have been created with refresh rates up to 28 Hz. However, the increased speed leads to reduced reliability. Therefore, for the purpose of expediting the demonstration of simulated virtual images, we reduced the frame rate to 12 Hz, which allows us to perform our demonstration with only limited flicker effects.

The monocular system employed in this experiment is convenient for proving the utility of perspective projection. However, it obscures somewhat the fact that focus cues, such as accommodation and vergence, and stereoscopic cues will be in conflict with other visual cues for the proposed “backdrop” voxels (see Visualization 5). In a binocular system, as the display scales, the solution to this conflict is the same for both types of conflicting cues: the observer distance to the voxels should be sufficiently large (the greater the distance, the better) to allow the other cues to dominate in visual influence over the accommodation, vergence, and stereoscopic cues. In the long term, we have proposed^7^ the use of particles with anisotropic scatter that can only be seen by one eye. In this way, two sets of particles (or one set, time-multiplexed) can be used to eliminate stereoscopic vergence and stereoscopic conflict.

The OTD drawing function was modified by perspective projection in synchronization with movement of the camera arm. The speed of panning was approximately 0.0194 m/s. Camera and lens used were a Canon EOS 6D and Canon MP-E 65 mm f/2.8 1–5× macro lens, respectively. The camera was focused at the chimney of the house (approximately *z* = 2 mm). The radius of swing was 100 mm to the front face of the camera lens. The house had dimensions of 7.7 × 10.6 × 7.4 mm. The moon had a diameter of 0.5 mm (which varied during the experiment). The moon was updated at 12 frames/s. The draw volume measured 0.5 mm in y and 9.2 mm in *x* (*z* volume was not used).

## Results

**Figure 3:**
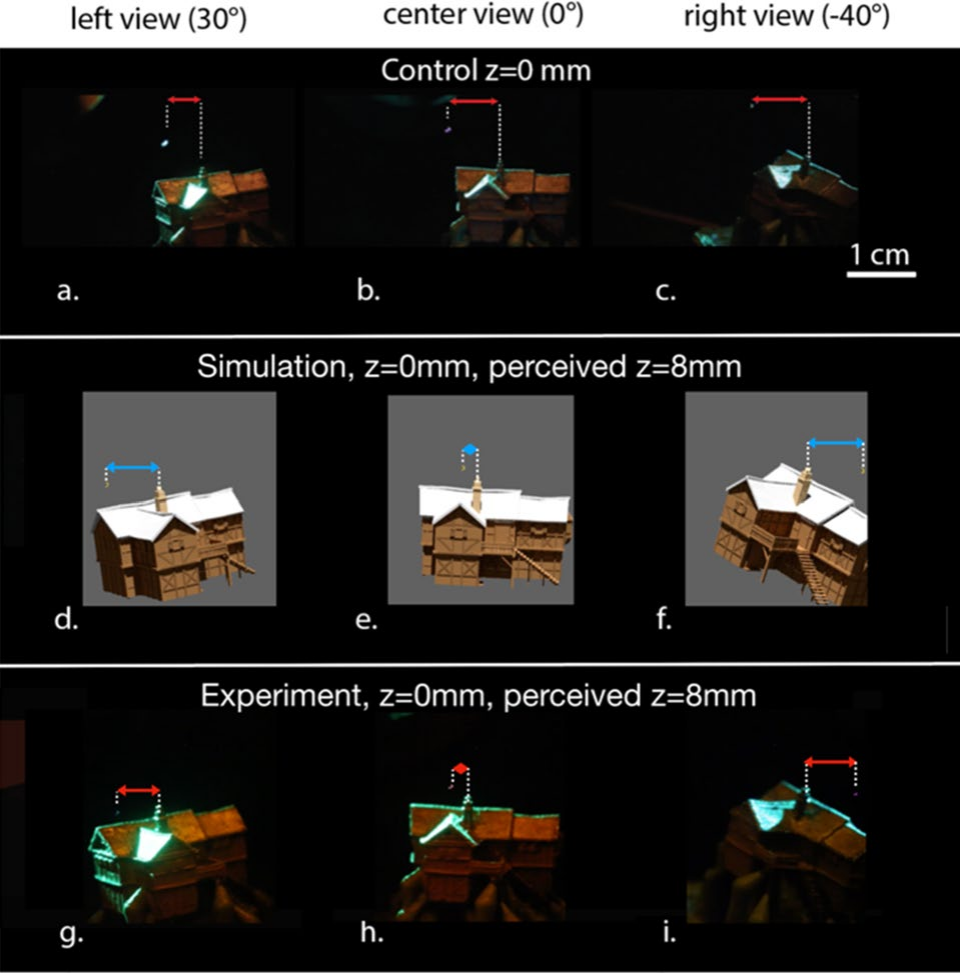
Experiment results. (**a**–**c**) Parallax for particle at z = 0 (in front of the house). (**d**–**f**) Simulation result, parallax for particle at z = 0, with perspective projection. (**g**–**i**) Experiment result, parallax for particle at z = 0, with perspective projection. The particle is in front of the house, but the parallax is consistent with a particle at z = 8 mm (behind the house). For full video, see “Supplemental Document” and Visualization 2.

In Fig. 3a–c, the moon is drawn in a plane in front of the house (flush with the front face at *z* = 0 mm). The moon is not modified as the camera rotates, providing a control image. In Fig. 3d–f, the moon is still drawn at *z* = 0, but the moon is shifted laterally as the camera rotates to give parallax consistent with an object perceived at *z* = 8 mm. In Fig. 3 g–i, the camera footage is superimposed over a Blender simulation (both with perspective projection activated). There is a bias due to imperfections in the setup, but the relative parallax agrees with simulation to within a 5.88% average error

4 $$error = \frac{(spc-epc)}{spc}\times 100$$

where *spc* is one component of the simulation pixel coordinate vector and *epc* is the corresponding component of the experimental pixel coordinate vector. Taking the Euclidean Average of the two error components gives an error of 5.88% in the image. In this experiment we increased the display space by 80% to 1.8 cm in one dimension compared to the physical volume of 1 cm^3^ of the display.

## Analysis

The modified parallax does appear to create images perceived behind the drawing volume. Our calculated error supports the use of this method. The modified parallax, after accounting for bias, shows good agreement with simulation. This shows the potential effectiveness of increasing the display space of the volumetric display beyond the physical boundaries of the display. The increase of display volume by 80% in one dimension demonstrated here can be extrapolated to infinity, given an immersive display where the viewer is always looking through the display volume.

Limitations of this approach include (1) a lack of binocular disparity, (2) requirement of motion tracking of the viewer’s eye position, and (3) mismatch of accommodation/vergence and other visual cues. To the first limitation, this experiment was a monocular test. To be effective for normal-sighted human viewers, our approach must eventually be modified to also provide accurate binocular parallax. For binocular parallax to function, the OTD must be capable of controllable anisotropic scatter. To date, we have demonstrated anisotropic scatter^9^ and we have outlined two possible methods for exerting control over this directional scatter in the future^9,12^ that would allow for each eye of the user to receive a different perspective based on their respective spatial locations. With the possible future addition of directional output control, the method proposed here would become more effective without any additional changes needed. The second limitation is that this method requires the viewer to be tracked (specifically the viewer’s head); this is a significant encumbrance as normal OTD real images require no knowledge of the user’s position and still provide almost 4π steradians of view angle. However, we can say that once directional scatter has been achieved, tracking of the viewer could be omitted in at least two dimensions (horizontal and vertical). The angular outputs of the display having image points corresponding to the perspective from that position updated regardless of viewer presence. The third dimension of the viewer position, the distance of the viewer from the display, would still be needed for ideal perspective reconstruction as the perspective projection is based on a 3D observation point. Further pursuit of directional scattering control is thus capable of solving one major shortcoming of OTD technology at this time, reducing the complexity of the method presented here, and extending the usefulness of the method presented here to include independent virtual images for several viewers at once. The final limitation is that of mismatch between the accommodative cue, which leads the user to focus at the projection plane, and the parallax cue, which leads the viewer to focus at the perceived point. This stereopsis/accommodation mismatch is common in other systems^13,14^ sometimes causing adverse side effects to users^15,16^. To mitigate it, we must place the perspective projection plane at a distance where parallax is more dominant than accommodation. This requirement is in harmony with the theatrical backdrop approach that we have proposed in this paper, especially given the relatively rapid drop-off of accommodation dominance with image distance^17^.

We would argue that, these limitations notwithstanding, simulating virtual images with OTD would be preferable to the use of a hybrid OTD/holography system, which has been proposed^3^. Unlike OTDs, holograms are extremely computationally intensive and their computational complexity scales rapidly with display size. The complexity also scales rapidly with point spread function. Neither is true for OTD displays. Consider a background of stars: regardless of the number of stars, a holographic display would require terabytes per second of data to provide the diffractive focusing power to render sharp star-like points, and the parallax and focus cues would be wasted given the extreme distance of the virtual points. By comparison, OTDs would only require a bandwidth proportional to the number of visible stars (1.8 Mb/s to represent the approximately 5000 visible stars)

5 $$\frac{data}{{sec}} = image\;points \times bytes\;per\;point \times frame\;rate$$

and would provide pinpoint acuity. Combined with the advantages of a single homogeneous display technology, there is a strong motivation to pursue simulated virtual OTD images.

Furthermore, we would argue that both volume images and perspective projection surfaces are best used in harmony and not to the exclusion of one or the other. For example, it would not be prudent to collapse the entirety of a scene, foreground and background, to a single plane and thereby lose the rare advantages of perfect accommodation and close-up viewing.

## Conclusion

We have demonstrated a display-level application for OTDs for the first time and have shown agreement with simulation to produce an effect similar to virtual images in optical trap displays. This result leads us to contemplate the possibility of immersive OTD environments that not only include real images capable of wrapping around physical objects (or the user themselves), but that also provide simulated virtual windows into expansive exterior spaces. See Fig. S1 in supplemental document and Visualization 4. This work provides a path forward in scaling the display content regardless of limitations in scaling the OTD physical display volume. The next steps in this work should include cues beyond parallax, such as occlusion and defocus. This work also strongly motivates the need for controllable directional scatter in OTD systems.

## Supplementary Information

Supplementary Information 1.

Supplementary Video 1.

Supplementary Video 2.

Supplementary Video 3.

Supplementary Video 4.

Supplementary Video 5.
