# Supplementary material for: Simulating virtual images in optical trap displays
Source: Sci Rep. 2021 Apr 6;11:7522. doi: 10.1038/s41598-021-86495-6 (PMC8024254; doi:10.1038/s41598-021-86495-6)
Supplement: Supplementary file 1 — Supplementary Information 1. [file 41598_2021_86495_MOESM1_ESM.doc]

Simulating virtual images in Optical Trap Displays: Supplementary Information

Wesley Rogers,^1^ and Daniel Smalley^1^ *

^1^Brigham Young University, Department of Electrical and Computer Engineering, Provo, UT 84602

*Corresponding author: smalley@byu.edu

Description of Visualization Materials:

**Visualization 1** shows a simulation of an OTD moon image with perspective projection at different depths.

**Visualization 2** gives video footage of the house/moon simulation and experiment illustrated in Figures 3 d-f and 3 g-i. The moon drawn in this example is *physically* in front of the house but perceived to be behind it.

**Visualization 3** shows a simulation of a more complex scene combining both real foreground images and simulated virtual images with a rotating perspective projection plane.

**Visualization 4** shows a simulation of how perspective projection could use OTD imagery projected inside a room to simulated images outside a room (a moon seen through a window).

**Visualization 5** shows video footage of from on moving observer and from a fixed, ‘witness’ camera for image depths of zero meters without perspective projection and 8 mm (800 units for life-size scale) with perspective projection. Because the moving camera rig occludes the fixed camera view for much of the video, we have added a green tracking bar to show the movement of the image when the image not visible.


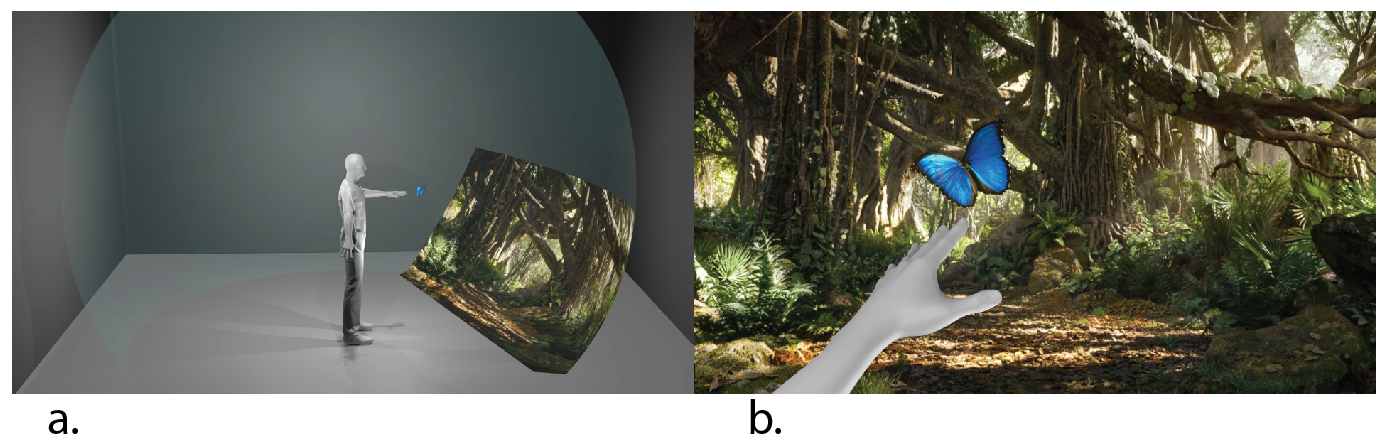


Fig. S1. Future OTD Backdrop concept. **a.** Third-Person View of an environment with both real OTD images and an OTD background for simulated virtual images. **b.** First person view of the scene in panel a.
